# Aggressive Behavior in Adolescents and Emerging Adults: The Psychometrics of the Portuguese Brief Peer Conflict Scale (Brief-PCS)

**DOI:** 10.3390/bs15101378

**Published:** 2025-10-10

**Authors:** Paula Vagos, Pedro F. S. Rodrigues, Josefa N. S. Pandeirada, Monica A. Marsee

**Affiliations:** 1 Department of Education and Psychology, University of Aveiro, 3810-193 Aveiro, Portugal; 2 William James Center for Research (WJCR), 3810-193 Aveiro, Portugal; 3 Center for Research in Neuropsychology and Cognitive and Behavioral Intervention (CINEICC), Faculty of Psychology and Educational Sciences, University of Coimbra, 3000-115 Coimbra, Portugal; 4 CINTESIS.UPT@RISE-Health, Portucalense University, 4200-072 Porto, Portugal; prodrigues@upt.pt; 5 Department of Psychology, Iowa State University, Ames, IA 50011, USA

**Keywords:** brief peer conflict scale, aggressive behavior, adolescence, emerging adulthood, sex, internal validity, convergent validity, confirmatory factor analysis

## Abstract

The Brief Peer Conflict Scale (Brief-PCS) has been shown to be psychometrically suitable for assessing the combination of the forms and functions of aggressive behavior in adolescence. However, its validity, invariance, and utility across other age groups remains unexplored. The current study aims to evaluate the psychometric properties of the Brief-PCS in community samples of adolescents and emerging adults, and to compare self-reported aggression across these age groups and by sex. A sample of 891 individuals (58.4% female, Mage = 16.69) completed the Brief-PCS and additional measures assessing psychopathy characteristics, forms of aggression, and overall aggression. Confirmatory factor analyses supported the four-factor measurement model (i.e., proactive overt, reactive overt, proactive relational, and reactive relational aggression) as the best fit for the data. Evidence also supported the scales’ internal consistency and convergent validity. This four-factor measurement model proved to be invariant across age groups and sex. Males reported being overall more aggressive than females, and adolescents reported more aggressive behaviors than emerging adults, except for proactive relational aggression. These findings extend prior research by confirming the Brief-PCS as a psychometrically sound and developmentally invariant tool, enhancing its value for examining both theoretical and applied aspects of aggression throughout the lifespan.

## 1. Introduction

Aggressive behavior is an intentional act that has the potential to cause harm to others who perceive the act as damaging (personally, in their possessions, and/or in their relationships; Archer & Coyne, 2005; Cohen et al., 2006). Such harm can assume diverse forms (i.e., the actual behavior that is practiced) and functions (i.e., the purpose or motivation behind the aggressive behavior; Cohen et al., 2006; Fite et al., 2023; Little et al., 2003). With regard to the forms of aggression, behaviors can be practiced in a direct or overt form (e.g., physical or verbal aggression), or in a covert and indirect or relational form (e.g., gossiping or excluding others; Archer & Coyne, 2005; Cohen et al., 2006). Regarding the functions of aggression, behaviors may be practiced proactively to achieve pre-defined goals, or they may be a reactive action to the perception of having been wronged or provoked in some way (Little et al., 2003).

The forms and functions of aggression may be combined to express four types of aggressive acts (i.e., reactive overt, proactive overt, reactive relational, and proactive relational aggression), but these combinations have seldom been addressed in a simplified manner by using one single assessment instrument, nor have they been considered across different age groups. The Peer Conflict Scale was designed specifically to assess these four constructs using 40 self-reported items and has been psychometrically appraised in community samples of north American (Marsee et al., 2011) and Portuguese adolescents (Vagos et al., 2014), as well as Portuguese young adults (Vagos et al., 2021). All those studies used confirmatory factor analyses and found evidence favoring a four-factor measurement model assessing reactive overt, proactive overt, reactive relational, and proactive relational aggression. Moreover, evidence was found for convergent validity in relation to psychopathy dimensions and delinquency (Marsee et al., 2011), and for sex-based measurement invariance (Vagos et al., 2014; Vagos et al., 2021). A brief 20-item version of the Peer Conflict Scale (Brief-PCS) has also been investigated within north American (Russell, 2014) and Portuguese community youth (Pechorro et al., 2021), as well as Portuguese detained youth (Pechorro et al., 2018). Again, all the works that investigated the psychometric properties of the Brief-PCS used confirmatory factor analyses and found evidence to support the applicability of the same four-factor measurement model proposed for the 40-item version of the instrument. Evidence also favored the convergent validity of the Brief-PCS, based on associations with psychopathic dimensions, impulsiveness, delinquency (Pechorro et al., 2021), or aggression (Pechorro et al., 2018). Moroń et al. (2023) more recently examined a modified 20-item version of the Peer Conflict Scale in an adult Polish sample. The same four-factor structure was replicated using confirmatory factor analyses. However, this instrument was based on a modified version that consisted of items selected from the 40-item version according to their loading values within that Polish sample; this modified version thus differs from the original instrument previously examined in north American and Portuguese adolescent samples. Hence, though the brief version of the Peer Conflict Scale may be useful to assess aggression, there is currently no evidence to attest for its applicability across diverse age groups (e.g., adult samples), as those groups have been considered using different versions of the Brief-PCS. This precludes assessment of the forms and functions of aggression invariantly across age groups, which is a key requirement for sound cross-group comparisons (Dimitrov, 2010), both cross-sectionally and longitudinally.

Accurate cross-age comparisons of aggression can lead to evidence-based insights into the developmental pathways of aggression, which, in turn, may point to specific vulnerability groups. Adolescents (i.e., individuals aged 12 to 20 years old) may be a particularly vulnerable group for increased practices of aggression, because their focus on self-discovery and identity development can make it harder for them to accept criticism and tolerate authority figures, and these difficulties may lead to unconventional or oppositional behaviors. On the other hand, emerging adults (i.e., individuals aged 18 to 29 years old) focus on finding their place within the broader society and on taking responsibility for their choices for the future, making them more likely to abide by social norms and rules (Feldman, 2019). In light of these elements, one could expect differences in the intensity and/or frequency of practicing aggression between the adolescent and emergent adult age groups. Nonetheless, previous works on the trajectories of aggression have not considered samples transitioning from adolescence to emerging adulthood, nor have they considered the forms (particularly the relational form) and functions of aggression (especially the reactive function; for a review see Jennings & Reingle, 2012).

Regarding the forms of aggression, it has been proposed that overt aggression tends to decline as individuals become more aware of social norms that disapprove of such behavior; instead, individuals might engage in more indirect and relational forms of aggression as they develop the verbal and social abilities necessary to effectively regulate social situations (Ingram, 2014). As for the functions of aggression, one previous study with male juvenile offenders found that reactive aggression steadily decreased from adolescence to emerging adulthood, whereas proactive aggression followed a low and stable course (Vaughan et al., 2023). These trajectories may differ depending on the individuals’ sex: for overt (i.e., physical and verbal) aggression, the differences between male and female individuals seems to widen over the life-course, with men being more overtly aggressive than women, and this gap reaches its peak by emerging adulthood. In contrast, women seem to resort more to indirect or relational forms of aggression during younger years, but the sex difference is not as pronounced in emerging adulthood (Côté, 2007). Previous works considering the combination of the forms and functions of aggression have reported mixed findings, namely that male adolescents and young adults engage more in those behaviors compared to females (Vagos et al., 2014, 2021), or that adolescent boys practice more proactive and reactive overt aggression, whereas girls score higher on reactive relational aggression (Marsee et al., 2011). Other studies have only found sex-based differences for proactive overt aggression as reported by adults (Moroń et al., 2023). To our best knowledge, no previous studies have compared self-reported aggression based on its forms and functions between adolescents and emerging adults, while also considering sex. Sex-based comparisons would, again, require invariance of measuring aggression using the Brief-PCS, something that has not been tested.

### 1.1. Goals and Hypotheses of the Present Study

Instruments that accurately assess the forms and functions of aggression within adolescents and emerging adults are currently scarce or unavailable; having such an instrument would allow for meaningful comparisons between and throughout those developmental stages. To address this research gap, the present study pursued two goals.

#### 1.1.1. Primary Goal

This work’s primary goal was to investigate the psychometric properties of the Brief-PCS, namely its factor structure, group measurement invariance, internal consistency, and convergent validity, in adolescent and emerging adult samples. Regarding convergent validity, and based on previous research that considered similar variables, we intended to test the association between the forms and functions of aggression and psychopathic dimensions (i.e., grandiose-manipulative, callous-unemotional, and impulsive-irresponsible), other aggression measures that consider its forms (relational, reputacional, overt, physical and verbal), and its emotional (i.e., anger) and cognitive (i.e., hostility) aspects. Considering the present work’s primary goal, our hypotheses were as follows:

PG1. Evidence will favor a four-factor internal structure that represents the combinations of the forms and functions of aggression for both age groups, similar to previous research with adolescents (e.g., Pechorro et al., 2021) and adults (e.g., Moroń et al., 2023).
PG2. The four-factor measurement model will be invariantly applicable to both age groups and to participants from both sexes, given that the 40-item version of the Peer Conflict Scale achieved sex-based measurement invariance within Portuguese adolescent (Vagos et al., 2014) and young adult (Vagos et al., 2021) samples.
PG3. Psychopathic characteristics (i.e., grandiose–manipulative, callous-unemotional, and impulsive-irresponsible) will be positively associated with aggression, more strongly with proactive relational and overt aggression (Nouvion et al., 2007; Pechorro et al., 2021); specifically, overt aggression will positively correlate with the affective dimension of psychopathy (i.e., callous–unemotional) whereas relational aggression will correlate with the interpersonal dimension (i.e., grandiose–manipulative; Vaughan et al., 2023).
PG4. Measures of reputational and relational aggression will be more highly intercorrelated, as will measures of overt, physical and verbal aggression (Pechorro et al., 2018; Queirós & Vagos, 2016).
PG5. Reactive aggression in both its forms will be positively associated with anger and hostility (Pechorro et al., 2018; Ramírez & Andreu, 2006; White & Turner, 2014).

#### 1.1.2. Secondary Goal

The secondary goal, which is dependent on the Brief-PCS psychometric appraisal, intends to analyze mean-level differences in self-reported aggression across age groups and sexes. Previous literature has seldom addressed adolescent and emerging adult age groups (for a review see Jennings & Reingle, 2012), particularly concerning the effect of both age and sex on the level of self-reported practice of aggression. We will add to that literature by specifically investigating sex mean-level differences across (i.e., female adolescents vs. female emerging adults and male adolescents vs. male emerging adults) and within age groups (i.e., female vs. male adolescents and female vs. male emerging adults). Concerning the present works’ secondary goal, our hypotheses were as follows:

SG1. Adolescent and emerging adult males (Vs. females) will score higher on all types of aggression measured by the Brief-PCS, consistent with findings from another sample examined by Vagos et al. (2014, 2021) that is culturally similar to the one used in the present study.
SG2. Regarding age-group comparisons, and because no work has previously compared adolescents and emerging adults on the forms and functions of aggression, our hypothesis is inferential. Given that proactive aggression diminishes while reactive aggression remains stable from 15 to 22 years old (Vaughan et al., 2023), and that overt aggression is gradually replaced by relational aggression (Ingram, 2014), we hypothesize that adolescents will report more proactive and overt aggression in comparison with emerging adults.

## 2. Materials and Methods

### 2.1. Participants

The initial sample included 923 participants, of which 18 were excluded based on being 30 years old or older. Another 14 participants were excluded due to presenting missing values; these accounted for only 1.55% of cases. A listwise approach was adopted because participants with or without missing values had similar mean ages [*t*(903) = −1.84, *p* = 0.07], and males and females were similarly distributed considering the presence/absence of missing values [*χ*^2^(2) = 5.07, *p* = 0.08].

The final complete sample was composed of 891 Portuguese males (*n* = 370, 41.5%) and females (*n* = 520, 58.4%), aged between 12 and 25 years old (*M* = 16.69, *SD* = 2.97). The complete sample included adolescent (aged 12 to 17 years old) and emerging adult (aged 18 to 25 years old) participants; see Table 1 for details on the adolescent and emerging adult samples. The adolescent sample included 477 participants; male and female adolescents’ mean age did not differ significantly [*t*(475) = 1.55, *p* = 0.12]. The emerging adult sample included 414 participants aged 18–25 years; females were significantly older than males [*t*(411) = −2.18, *p* = 0.03], though descriptive means were very close. Males and females were not evenly distributed among age groups [*χ*^2^(1) = 25.05, *p* < 0.001]; specifically, females were overrepresented in the emerging adulthood sample whereas males were more prevalent than statistically expected in the adolescent sample.

### 2.2. Instruments

All instruments were used in their European Portuguese versions, and all data were collected using a paper and pencil format. The Brief-PCS was applied to both the adolescent and the emerging adult samples. The Youth Psychopathy Inventory–Short and the Revised Peer Experience Questionnaire were applied only to adolescents, namely subsample 1 (*n* = 191) and subsample 2 (*n* = 283), respectively (see Table 1). The Buss-Perry Aggression Questionnaire was applied only to the subsample of emerging adults (i.e., Subsample 3, *n* = 337; see Table 1).

#### 2.2.1. Brief Peer Conflict Scale (Brief-PCS)

The Peer Conflict Scale was developed to assess the combination of the forms and functions of aggression: reactive overt, proactive overt, reactive relational, and proactive relational aggression. Previous studies confirmed a four-correlated factors internal structure in adolescent (Marsee et al., 2011) and young adults’ samples (Vagos et al., 2021) for its 40-item version. Those measures have also attained good internal consistency values (Marsee et al., 2011; Vagos et al., 2021) and were associated with other relevant variables, such as psychopathic characteristics or delinquency (Marsee et al., 2011). The Brief-PCS takes 20 from the original 40 items. Those 20 items were selected based on conceptual and empirical motives: items were selected if their content prototypically represented the concept of each of the combinations of the forms and functions of aggression and if they had relevant item-total correlation and loading values onto their allocated measure (Scott et al., 2014). Previous research found those 20 items to be organized into the same four-factor measurement model, in community (Pechorro et al., 2021; Russell, 2014) and detained adolescent samples (Pechorro et al., 2018). Moreover, measures achieved at least good internal consistency indicators: α ≥ 0.72 for detained youth (Pechorro et al., 2018) and α ≥ 0.83 for community adolescents (Pechorro et al., 2021). Evidence for construct validity was also found in relation to psychopathic characteristics, self-reported delinquency, lack of self-control (Pechorro et al., 2021), and self-esteem (Pechorro et al., 2018).

We applied the Portuguese version of Brief-PCS developed by Vagos et al. (2014, 2021) and used the 20 items proposed by Scott et al. (2014) and Russell (2014). Responses were given using a Likert-type scale ranging from 1 (it has nothing to do with me) to 4 (it has everything to do with me), with higher scores indicating higher self-reported practice of aggressive behavior. The psychometric properties of this instrument using the current samples are presented in the Results section.

#### 2.2.2. Youth Psychopathy Inventory–Short Version

This inventory includes 18 items aimed at measuring three dimensions of psychopathic characteristics in adolescents: grandiose-Manipulative (i.e., interacting via dishonest charm, lying or manipulating others while holding a grandiose self-perception), callous-unemotional (i.e., feeling remorselessness, unemotional and manifesting callousness), and impulsive-orresponsible (i.e., acting towards thrill-seeking in an impulsive or irresponsible way). Respondents rate each item using a Likert-type response scale ranging from 0 (does not apply at all) to 3 (applies very well). Previous psychometric findings revealed the instruments’ factorial validity, good internal consistency, and construct validity in relation to conduct problems (van Baardewijk et al., 2010), self-reported aggression, and lack of empathy (Pechorro et al., 2017). Its three dimensions achieved good to very good internal consistency values using adolescent subsample 1 (*n* = 191): α = 0.80 for grandiose-manipulative, α = 0.72 for callous-unemotional, and α = 0.73 for impulsive-irresponsible.

#### 2.2.3. Revised Peer Experience Questionnaire

This questionnaire uses 28 items to assess three forms of aggressive behavior (i.e., overt, relational, and reputational) and prosocial behavior, as it is perpetuated (i.e., bully version) and received (i.e., victim version; Prinstein et al., 2001). Participants rate, for each item, how often they engaged in or were the recipients of aggressive or prosocial behavior in the past year. Responses are provided using a 5-point Likert-type scale ranging from 1 (never) to 5 (a few times a week). Previous evidence supports a four-factor measurement model for both the bully and the victim versions, good internal consistency values for those measures (Prinstein et al., 2001; Queirós & Vagos, 2016), and construct validity in relation to psychopathic characteristics and to poor attachment to parents and peers (Queirós & Vagos, 2016). Only the bully version and the three forms of aggression were considered in the current work given our goals, namely overt aggression, relational aggression (i.e., damaging others’ relationships by manipulation or exclusion) and reputational aggression (i.e., damaging others’ relationships by lying or gossiping to diminish their social status). The internal consistency values for those measures obtained using our adolescent subsample 2 (*n* = 283) were α = 0.78 for overt aggression, α = 0.77 for reputational aggression, and α = 0.58 for relational aggression.

#### 2.2.4. Buss-Perry Aggression Questionnaire

This instrument consists of 29 items designed to assess physical aggression, verbal aggression, anger, and hostility. Items are rated using a Likert-type scale that varies between 1 (never or almost never) to 5 (always or almost always). Previous evidence favors the four-factor measurement model measuring physical aggression, verbal aggression, anger and hostility. Adequate internal consistency was also reported for those measures (Buss & Perry, 1992; Cunha & Gonçalves, 2013). Using the data from our emerging adult subsample 3 (*n* = 337), we found the following internal consistency values: α = 0.66 for physical aggression, α = 0.62 for verbal aggression, α = 0.57 for anger, and α = 0.78 for hostility.

### 2.3. Procedures

#### 2.3.1. Recruitment Procedures

The necessary authorizations were obtained to conduct the study in Public Schools and Public Higher Education institutions from the North and Center regions of Portugal. For the adolescent sample, we requested authorization from the General Directory for Education to gather data within public schools. We also asked the schools to collaborate by sharing the study with students and their legal guardians. Then, informed consent forms were sent to legal guardians and students. For the emerging adult sample, Higher Education teachers were asked to make time available in their class so that the research team could present the study and invite for participation; informed consent was obtained from all participants prior to participation In both cases, students filled in the self-report protocol in class, using time made available by the teacher, and only after the applicable authorizations and informed consents were obtained. No compensation was given to participants for taking part in this study.

To test for convergent validity in relation to external variables, two subsamples of adolescents responded to one additional questionnaire each to minimize participant burden; these subsamples consisted of students within randomly selected classes. Specifically, 191 adolescents filled in the Youth Psychopathy Inventory–Short (subsample 1), and another 283 adolescents filled in the Revised Peer Experience Questionnaire (subsample 2). A subsample of 337 emerging adults, also selected randomly based on class, responded to the Buss-Perry Aggression Questionnaire in addition to the Brief-PCS (subsample 3). See Table 1 for details on these subsamples.

#### 2.3.2. Statistical Analyses

All data analyses presented in this manuscript were carried out in April 2025. Confirmatory Factor Analyses and Measurement Invariance Analyses were carried out using the Maximum Likelihood Robust estimator because the data were not multivariate normal (Mardia’s multivariate skewness = 447.84 and Mardia’s multivariate kurtosis = 1494.24, both with *p* < 0.001). The fit of the measurement models was judged considering the guidelines provided by Hu and Bentler (1999): fit was considered acceptable based on finding a Standardized Root Mean Square Residual (SRMR) < 0.09, combined with either a Root Mean Square Error of Approximation (RMSEA) < 0.06 or with a Comparative Fit Index (CFI) > 0.95. Measurement invariance was tested using a forward approach (Dimitrov, 2010) and sequential multiple-group CFAs, in which the measurement model was judged based on its fit for each independent group (i.e., configural invariance), on its comparative fit when restraining loading values to be equal across groups (i.e., metric invariance), and then on its comparative fit when adding an equality constraint to the items’ intercepts (i.e., scalar invariance). When factor loadings and indicator intercepts are invariant across groups, strong measurement invariance is established, and meaningful comparisons of latent factor means across groups are deemed acceptable (Dimitrov, 2010). Metric invariance was judged based on ∆CFI ≤ −0.01 combined with ∆RMSEA ≤ 0.015 or ∆SRMR ≤ 0.03 when comparing an unrestricted model (i.e., no equality constraints) to the loading equality constraint model. Scalar invariance was considered based on ∆CFI ≤ −0.01 combined with ∆RMSEA ≤ 0.015 or ∆SRMR ≤ 0.01when comparing the loading equality constraint model to the loading and intercept constraint model (Chen, 2007).

Following a minimal percentage of measurement variance (less than 20% freed parameters; Dimitrov, 2010), we analyzed the effects of age-group and sex on the mean values of self-reported aggressive behavior using two-way independent ANOVAs. Internal consistency was analyzed via the Cronbach Alpha. Values above 0.60 were considered acceptable given the low number of items composing each measure (Streiner, 2003). Convergent validity in relation to external variables relied on non-parametric two-tailed correlation analyses between measures, using the Spearman Rho estimator.

Analyses were conducted using licensed versions of SPSS v.21 (i.e., two-way independent ANOVAs, correlation analyses) or Mplus v.7.4 (Muthén & Muthén, 1998–2017; i.e., confirmatory factor analyses, measurement invariance analyses), which fully supported the procedures applied in this study. These procedures are implemented identically in current software versions, ensuring that the results are valid and fully reproducible.

## 3. Results

### 3.1. Measurement Model

As carried out in previous works for the complete (Marsee et al., 2011; Vagos et al., 2014, 2021) and brief versions of the Peer Conflict Scale (Moroń et al., 2023; Pechorro et al., 2018, 2021), we compared four competing measurement models for the Brief-PCS using the complete sample (i.e., including adolescent and emerging adult participants; *n* = 891): (1) a unidimensional model including all items; (2) a two-factor model depicting the forms of aggression (i.e., relational and overt); (3) a two-factor model portraying the functions of aggression (i.e., proactive and reactive); and, (4) a four-factor model representing four combinations of the forms and functions of aggression (i.e., reactive overt, proactive overt, reactive relational, and proactive relational). Models 2 and 4 were a good fit for the data (cf. Table 2), thus partially confirming hypothesis PG1. Model 4 was selected for further analyses motivated by theoretical (i.e., higher diversity of constructs) and statistical reasons (i.e., better fit indices).

**Figure 1:**
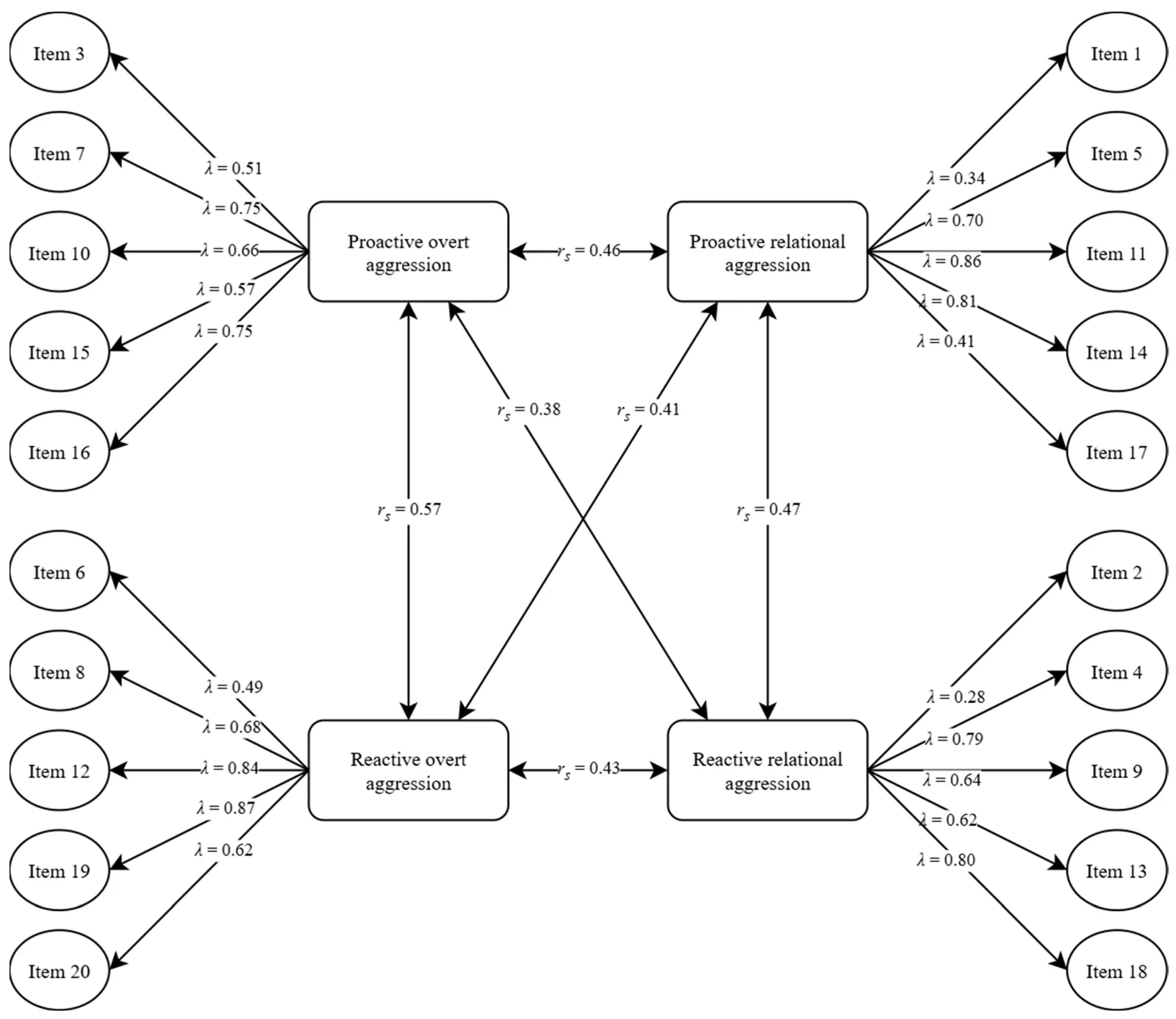
Item loading values and spearman correlation values between measures taken from Model 4: 20-item four-factor forms and functions of aggression model. Note: All loading and correlation values were significant at *p* < 0.001.

Loading values were always significant for the 20-item four-factor model (*p* < 0.001) and varied between 0.49 and 0.87 for reactive overt aggression, between 0.51 and 0.75 for proactive overt aggression, between 0.28 and 0.80 for reactive relational aggression, and between 0.34 and 0.86 for proactive relational aggression. The four measures taken from this model correlated positively and significantly, ranging from *r_s_* = 0.38, *p* < 0.001 between reactive overt and proactive relational aggression, to *r_s_* = 0.57, *p* < 0.001 between reactive and proactive overt aggression; see Figure 1 (for similar results concerning Model 2, see Figure S1 in the Supplementary Material). All measures taken from Model 4 achieved at least acceptable internal consistency values: α = 0.80 for reactive overt aggression, α = 0.76 for proactive overt aggression, α = 0.63 for reactive relational aggression, and α = 0.64 for proactive relational aggression. Model 4, including 20-items organized into four correlated factors, was carried forward to the age- and sex-based measurement invariance analyses.

### 3.2. Invariance of the Measurement Model

#### 3.2.1. Age-Based Measurement Invariance

Age-based measurement invariance considered the adolescent and emerging adult samples. The 20-item four-factor model was a good fit for both age samples taken separately, pointing to configural invariance (cf. Table 2). Full metric invariance was not found [ΔCFI = −0.024; ΔRSMEA = 0.0003; ΔSRMR = 0.027], meaning that not all item loading values may be considered invariant across groups. Partial metric invariance was achieved after relaxing the loading values for items 11 and 14 to vary across the adolescent and emerging adult samples [ΔCFI = −0.008; ΔRSMEA = 0.0000; ΔSRMR = 0.014]. Then, full scalar invariance was obtained [ΔCFI = −0.009; ΔRSMEA = 0.0001; ΔSRMR = 0.003], meaning that all item intercepts may be considered equal concerning the adolescent and emerging adult samples. Given that less than 20% of parameters were allowed to vary between age groups, findings sustain our hypothesis PG2 and point to meaningful age-based measurement invariance.

#### 3.2.2. Sex-Based Measurement Invariance

Sex-based measurement invariance considered the male and female participants within the complete sample (i.e., including adolescent and emerging adults). The 20-item four-factor model was a good fit for male and female participants taken separately (cf. Table 2) indicating configural invariance. Full metric invariance was not found [ΔCFI = −0.046; ΔRSMEA = 0.0007; ΔSRMR = 0.047], indicating that some items may have different loading values across groups. Instead, partial metric invariance was found after relaxing loading values for items 11, 14 and 18 so that they could vary between male and female participants [ΔCFI = −0.008; ΔRSMEA = 0.0000; ΔSRMR = 0.017]. Likewise, full scalar invariance was not found [ΔCFI = −0.011; ΔRSMEA = 0.0001; ΔSRMR = 0.005], meaning variant item intercept values across groups. Only partial scalar invariance was achieved after allowing the intercept of item 1 to vary between sexes [ΔCFI = −0.008, ΔRSMEA = 0.0001; ΔSRMR = 0.004]. Given that less than 20% of parameters were allowed to vary between sexes, findings sustain our hypothesis PG2 and point to meaningful sex-based measurement invariance.

### 3.3. Convergent Validity in Relation to Other Variables

Measures relating to psychopathic characteristics and to the practice of aggressive behavior correlated significantly and positively with the four measures of the Brief-PCS (cf. Table 3) thus sustaining our hypotheses PG3, PG4, and PG5. It is noteworthy that: (1) the grandiose-manipulative psychopathy dimension correlated the highest with proactive and relational measures of aggression, the callous-unemotional dimension had higher correlation values with the proactive and overt measures, and the impulsive-irresponsible dimension had higher correlation values with the reactive and relational measures; (2) overt aggression and physical aggression achieved higher correlation values with the overt measures of the Brief-PCS, whereas relational and reputational aggression achieved higher correlation values with the relational measures of the Brief-PCS; and, (3) anger correlated the highest with reactive overt aggression, whereas hostility correlated the highest with reactive relational aggression.

### 3.4. Effects of Age Groups and Sex on Self-Reported Practice of Aggressive Behaviors

Findings show a significant effect of sex for reactive overt [*F*(1,886) = 20.77, *p* < 0.001, *ƞp*^2^ = 0.023], proactive overt [*F*(1,886) = 25.07, *p* < 0.001, *ƞp*^2^ = 0.028], reactive relational [*F*(1,886) = 6.24, *p* = 0.013, *ƞp*^2^ = 0.007], and proactive relational aggression [*F*(1,886) = 23.98, *p* < 0.001, *ƞp*^2^ = 0.026]: males always scored higher than female, thus confirming our hypothesis SG1. There was also a significant effect of age for reactive overt [*F*(1,886) = 32.47, *p* < 0.001, *ƞp*^2^ = 0.035], proactive overt [*F*(1,886) = 9.29, *p* = 0.002, *ƞp*^2^ = 0.010], and reactive relational aggression [*F*(1,886) = 16.37, *p* = 0.001, *ƞp*^2^ = 0.018], but not for proactive relational aggression [*F*(1,886) = 0.16, *p* = 0.69]; adolescents always scored higher than emerging adults thus adding evidence in favor of our hypothesis SG2. The sex*age interaction was not statistically significant for any of the combinations of the forms and functions of aggression (lowest *p* > 0.22). See Table 4 for further details on descriptive values.

## 4. Discussion

Aggressive behavior has been proposed as a multidimensional construct, with diverse forms associated with its expression and different functions underlying its motives (Fite et al., 2023). The Brief-PCS has been psychometrically appraised as a way of assessing such multidimensionality in adolescents (Pechorro et al., 2018, 2021; Russell, 2014), while the full 40-item version of the Peer Conflict Scale has also been investigated in young adults (Vagos et al., 2021). In particular, the psychometric properties and measurement invariance across adolescents and emerging adults have not been tested for the Brief-PCS. Such study is essential to allow sound comparisons between developmentally diverse groups, who face different intrapersonal and contextual developmental challenges (Feldman, 2019) that may influence aggressive behaviors (Cohen et al., 2006). Also contrasting to the complete version of the Peer Conflict Scale (e.g., Vagos et al., 2014), sex-based measurement invariance had not been established for the Brief-PCS. The present work investigated the internal structure, internal consistency and convergent validity of the Brief-PCS as its primary goal. Additionally, we explored age- and sex-based mean-level differences on the self-reported practice of aggression, given the lack of prior comparisons between adolescence and emerging adulthood.

In terms of the internal structure of the Brief-PCS, current findings confirmed hypothesis PG1 and replicated the four-factor internal structure of reactive overt, proactive overt, reactive relational, and proactive relational aggression, similar to previous findings with adolescents (Pechorro et al., 2018, 2021; Russell, 2014) and young adults (Vagos et al., 2021). This provides further evidence for the utility of assessing both the forms and functions of aggression within one brief self-report measure and supports theoretical models positing that the form/function combinations are distinct constructs (Marsee et al., 2011). Likewise, this confirmation aligns with the forms (Card et al., 2008) and the functions (Khouwaga Yusoufzai & Lobbestael, 2022) of aggression having been associated differently with diverse outcomes. The four-factor model representing the combinations of the forms and functions of aggression was found to similarly (i.e., invariantly) represent aggression expressed by diverse age (i.e., adolescents and emerging adults) and sex groups (i.e., females and males), which aligns with our hypothesis PG2. Though experiencing different developmental changes and challenges, adolescents and emerging adults have developed a set of cognitive and interpersonal skills (Feldman, 2019); such skills may suggest that the same framework can be applied to assessing the aggressive behaviors of both age groups.

Our results regarding convergent validity confirm our hypothesis PG3, in as much as the four factors of the Brief-PCS were positively associated with psychopathic dimensions. Specifically, the interpersonal dimension (i.e., grandiose–manipulative, reflected in relating to others in a manipulative way to serve one’s purposes) correlated more strongly with proactive (Guerra & White, 2017) and with relational aggression (Verona et al., 2023); the affective dimension (i.e., callous-unemotional, meaning lack of empathy, guilt or remorse in relation to the consequences of ones’ acts on others) associated more strongly with overt aggression (Verona et al., 2023), and the behavioral dimension (i.e., an impulsive-irresponsible way of acting without prior pondering), associated more strongly with reactive aggression (Guerra & White, 2017). Furthermore, and sustaining our hypothesis PG4, measures of the forms of aggression of the Brief-PCS (irrespective of their functions) associated in the predicted manner with other measures of aggression: relational aggression (Vs. overt aggression) correlated higher with relational and reputational aggression measured by the Revised Peer Experience Questionnaire (Queirós & Vagos, 2016), and overt aggression (Vs. relational aggression) correlated higher with physical and verbal aggression measured by the Buss-Perry Aggression Questionnaire (Cunha & Gonçalves, 2013). Finally, and favoring our hypothesis PG5, anger was more strongly associated with reactive measures (Vs. proactive measures of the same form; Ramírez & Andreu, 2006; White & Turner, 2014). The highest correlation value found for hostility was with reactive relational aggression. This form of aggression often involves careful planning, including identifying potential accomplices (Warren et al., 2011). Such planning may reflect the cognitive aspects of hostility, specifically a persistent negative attitude and resentment toward others (Christopher et al., 2024) which can ultimately culminate in aggressive acts of retaliation.

Concerning the mean-level practice of aggression, comparisons within each age-group revealed that male participants reported more aggression (for all its measures) than female participants, which is consistent with previous findings using the Brief-PCS within adolescent (Pechorro et al., 2021) and young adult community samples (Vagos et al., 2021). This finding confirms our hypothesis SG1 and adds evidence to the sex-based gap in the practice of overt and relational aggression (Côté, 2007) while placing its beginning during adolescent years. As for the age-group comparison, and partially confirming our hypothesis SG2, adolescent participants scored higher than emerging adults in proactive overt aggression and in both reactive overt and relational aggression, but not in proactive relational aggression. In comparison with the male young offender sample used by Vaughan et al. (2023) who found a stable course for reactive but not for proactive aggression, our comparisons based on a cross-sectional design and on community samples suggest that normative developing individuals develop the tools to better manage their behavior in relation to social norms, thus engaging in behaviors that enable them to avoid punishment and/or fit within the broader society (Feldman, 2019). This may explain a general decrease in the practice of overt aggression and of reactive aggression seen from adolescence to emerging adulthood. Such patterns also reflect what Moffitt (1993) described as the adolescent-limited developmental course for aggression, which was found in most studies reviewed by Jennings and Reingle (2012) to be more common. Within that developmental course for aggression, aggression declines as individuals experience relevant life events like attending college (Moffitt, 1993), and such experiences tend to occur precisely in emerging adulthood (Feldman, 2019). Adolescents and emerging adults did not differ in the practice of proactive relational aggression (i.e., the use of relational aggression in a pondered way to achieve one’s goals). This may be because this particular behavior may serve the same adaptive and developmental goals across sexes and age-groups, as it allows both males and females to exert dominance in a way that is, seemingly, cooperative and not openly punished by most human societies (Ingram, 2014). Interestingly, we found no significant interactions between sex and age, indicating that males and females’ trajectories are similar across adolescence and emerging adulthood, when considering the combination of the forms and functions of aggression. This aligns with common findings on the developmental trajectories of violence, aggression, and delinquency as reviewed by Jennings and Reingle (2012) that are mostly shared by male and female participants.

Though promising, the current work is not without limitations. We did not use a longitudinal design. Even if our findings advance knowledge by comparing age groups that have scarcely ever been compared before (see Jennings & Reingle, 2012 for a review), they do not portray developmental profiles or trajectories. Nonetheless, current findings suggest that the Brief-PCS is a psychometrically sound instrument to extend the investigation considering the transition from adolescence to later developmental stages, including emerging adulthood. Finally, the evidence on the convergent validity of the Brief-PCS, though compelling, was based solely on self-report instruments and the internal consistency of the relational aggression and anger measures were lower than desirable; additionally divergent validity was not considered in this work. Though self-report instruments have been found to accurately distinguish the functions of aggression (Polman et al., 2007), resorting to other forms of assessing aggression (e.g., peer nomination, Mehari et al., 2019) should be considered in future studies to provide further evidence on the construct validity and applied utility of the Brief-PCS.

## 5. Conclusions

The current work provides robust evidence that the Brief-PCS is a psychometrically sound instrument for assessing the forms and functions of aggression. Results supported a four-factor structure (reactive overt, proactive overt, reactive relational, and proactive relational aggression) that was invariant across developmental stages (adolescents and emerging adults) and sexes, extending previous validation efforts in Portuguese and international samples (e.g., Pechorro et al., 2018, 2021; Moroń et al., 2023). Furthermore, the Brief-PCS was sensitive to expected mean-level differences, with males scoring higher than females and adolescents reporting more overt and proactive aggression than emerging adults. These results are consistent with developmental research showing age- and sex-related variations in aggression (Ingram, 2014; Vaughan et al., 2023). By demonstrating both structural validity and sensitivity to group differences, this work strengthens confidence in the Brief-PCS as a reliable tool for advancing research on the developmental course of aggression and for enabling meaningful cross-group comparisons. Future studies should replicate these findings in more diverse cultural contexts and explore longitudinal applications to further establish the instrument’s utility in developmental and applied research.

## Figures and Tables

**Table 1:** Sample and subsample characterization, based on sex and age.

		N (Age Range)	M_age_ (SD)	Males		Females	
				N (%)	M_age_ (SD)	N (%)	M_age_ (SD)
Adolescent sample		477 (12–17)	14.30 (1.43)	235 (49.3)	14.40 (1.42)	242 (50.7)	14.20 (1.43)
	Subsample 1	191 (14–17)	15.54 (1.05)	112 (58.6)	15.46 (0.98)	79 (41.4)	15.67 (1.03)
	Subsample 2	283 (12–15)	13.48 (0.97)	120 (42.4)	13.46 (0.96)	163 (57.6)	13.49 (0.98)
Emerging adult sample		414 (18–25)	19.43 (1.5)	135 (32.6)	19.18 (1.58)	278 (67.1)	19.54 (1.56)
	Subsample 3	337 (18–25)	19.76 (1.59)	96 (28.6)	19.66 (1.65)	240 (71.2)	19.78 (1.54)

Note: One emerging adult participant did not provide information on their sex. Subsample 1 filled in the Brief-PCS and the Youth Psychopathy Inventory—Short. Subsample 2 filled in the Brief-PCS and the Peer Experience Questionnaire. Subsample 3 filled in the Brief-PCS and the Buss-Perry Aggression Questionnaire.

**Table 2:** Measurement model and measurement invariance analyses.

		*χ* ^2^	df	RMSEA	90% CI for RMESEA	CFI	SRMR
Measurement model (*n* = 891)							
	Model 1: 20-item one-factor model	906.84	170	0.070	0.065; 0.074	0.78	0.077
	Model 2: 20-item two-factor forms of aggression model	581.85	169	0.052	0.048; 0.057	0.88	0.072
	Model 3: 20-item two-factor functions of aggression model	2310.09	169	0.119	0.115; 0.124	0.75	0.077
	Model 4: 20-item four-factor forms and functions of aggression model	459.41	164	0.045	0.040; 0.050	0.91	0.064
Age-based measurement invariance							
	Adolescent sample (*n* = 477)	401.79	164	0.055	0.048; 0.062	0.90	0.070
	Emerging adult sample (*n* = 414)	328.82	164	0.049	0.042; 0.057	0.79	0.079
	Unconstraint model	732.91	328	0.053	0.048; 0.058	0.87	0.074
	Full metric invariance model	824.79	344	0.056	0.051; 0.061	0.85	0.101
	Partial metric invariance model	771.71	342	0.053	0.048; 0.058	0.87	0.088
	Full scalar invariance model	817.55	358	0.054	0.049; 0.059	0.86	0.091
Sex-based measurement invariance							
	Male participants (*n* = 370)	308.63	164	0.049	0.040; 0.057	0.94	0.058
	Female participants (*n* = 520)	370.56	164	0.049	0.043; 0.056	0.80	0.074
	Unconstraint model	686.69	328	0.050	0.044; 0.055	0.89	0.068
	Full metric invariance model	847.50	344	0.057	0.042; 0.062	0.84	0.115
	Partial metric invariance model	726.35	341	0.050	0.045; 0.055	0.88	0.085
	Partial metric and full scalar invariance model	775.77	357	0.051	0.046; 0.056	0.87	0.090
	Partial metric and partial scalar invariance model	764.94	356	0.051	0.046; 0.056	0.087	0.089

**Table 3:** Spearman rho correlation values between the measures of the Brief-PCS and the external variables.

		Brief—Peer Conflict Scale (Brief-PCS)			
		Proactive Overt Aggression	Proactive Relational Aggression	Reactive Overt Aggression	Reactive Relational Aggression
Youth Psychopathy Inventory—Short					
	Grandiose-manipulative	0.27 ***	0.30 ***	0.22 ***	0.27 ***
	Callous-unemotional	0.24 ***	0.19 **	0.25 ***	0.21 ***
	Impulsive-irresponsible	0.22 ***	0.25 ***	0.28 ***	0.30 ***
Peer Experience Questionnaire—Revised					
	Overt aggression	0.45 ***	0.35 ***	0.52 ***	0.24 ***
	Relational aggression	0.33 ***	0.38 ***	0.32 ***	0.35 ***
	Reputational aggression	0.19 **	0.41 ***	0.18 *	0.34 ***
Buss-Perry Aggression Questionnaire					
	Anger	0.24 ***	0.22 ***	0.33 ***	0.21 ***
	Physical aggression	0.40 ***	0.34 ***	0.51 ***	0.24 ***
	Hostility	0.29 ***	0.23 ***	0.28 ***	0.32 ***
	Verbal aggression	0.31 ***	0.30 ***	0.35 ***	0.25 ***

Note: Only adolescent subsamples responded to the Youth Psychopathy Inventory—Short and the Revised Peer Experience Questionnaire. The Buss-Perry Aggression Questionnaire was filled in only by emerging adults. Two-tailed significance levels are reported. *** *p* < 0.001, ** *p* < 0.01, * *p* < 0.05.

**Table 4:** Descriptive data for measures of the Brief Peer Conflict Scale, for the complete sample and by age and sex groups.

	Complete Sample			Adolescent Sample			Emerging Adult Sample		
	Total (*n* = 891)	Females (*n* = 520)	Males (*n* = 370)	Total (*n* = 477)	Females (*n* = 242)	Males (*n* = 235)	Total (*n* = 414)	Females (*n* = 278)	Males (*n* = 135)
Proactive overt aggression	5.86 (1.91)	5.50 (1.29)	6.29 (2.49)	6.08 (2.32)	5.68 (1.51)	6.49 (2.88)	5.59 (1.25)	5.44 (1.05)	5.93 (1.54)
Proactive relational aggression	6.28 (1.99)	5.99 (1.44)	6.67 (2.52)	6.35 (2.30)	6.03 (1.49)	6.69 (2.87)	6.18 (1.56)	5.96 (1.39)	6.64 (1.77)
Reactive overt aggression	6.59 (2.76)	6.17 (2.39)	7.19 (3.12)	7.16 (3.31)	6.76 (3.08)	7.57 (3.49)	5.95 (1.75)	5.66 (1.38)	6.55 (2.22)
Reactive relational aggression	6.85 (2.01)	6.67 (1.62)	7.12 (2.44)	7.13 (2.39)	6.92 (1.87)	7.35 (2.81)	6.53 (1.39)	6.44 (1.33)	6.70 (1.51)

Note: Total refers to both male and female participants within each sample. Values are taken from the 20-item four-factor measurement model. Values are presented as M (SD).

## Data Availability

The data presented in this study are available on request from the corresponding author. The data are not publicly available due to privacy or ethical restrictions.

## Supplementary Materials

The following supporting information can be downloaded at https://www.mdpi.com/article/10.3390/bs15101378/s1: Figure S1: Item loading values and spearman correlation values between measures taken from Model 2: 20-item four-factor forms of aggression model.
