# Correction: Vagos et al. (2025). Aggressive Behavior in Adolescents and Emerging Adults: The Psychometrics of the Portuguese Brief Peer Conflict Scale (Brief-PCS). *Behavioral Sciences*, *15*(10), 1378

**DOI:** 10.3390/bs16081283

**Published:** 2026-07-28

**Authors:** Paula Vagos, Pedro F. S. Rodrigues, Josefa N. S. Pandeirada, Monica A. Marsee

**Affiliations:** 1 Department of Education and Psychology, University of Aveiro, 3810-193 Aveiro, Portugal; 2 William James Center for Research (WJCR), 3810-193 Aveiro, Portugal; 3 Center for Research in Neuropsychology and Cognitive and Behavioral Intervention (CINEICC), Faculty of Psychology and Educational Sciences, University of Coimbra, 3000-115 Coimbra, Portugal; 4 RISE-HEALTH@UPT, Portucalense University, 4200-072 Porto, Portugal; 5 Department of Psychology, Iowa State University, Ames, IA 50011, USA

## Values in Table

In the original publication (Vagos et al., 2025), there was a mistake in the mean and standard deviation values presented for the proactive relational aggression and reactive relational aggression measures displayed in Table 4. The correct version of Table 4 with the correct values appears below. The authors state that the scientific conclusions are unaffected. This correction was approved by the Academic Editor. The original publication has also been updated.

## Figures and Tables

**Table 4:** Descriptive data for measures of the Brief Peer Conflict Scale, for the complete sample and by age and sex groups.

	Complete Sample			Adolescent Sample			Emerging Adult Sample		
	Total (*n* = 891)	Females (*n* = 520)	Males (*n* = 370)	Total (*n* = 477)	Females (*n* = 242)	Males (*n* = 235)	Total (*n* = 414)	Females (*n* = 278)	Males (*n* = 135)
Proactive overt aggression	5.86 (1.91)	5.50 (1.29)	6.29 (2.49)	6.08 (2.32)	5.68 (1.51)	6.49 (2.88)	5.59 (1.25)	5.44 (1.05)	5.93 (1.54)
Proactive relational aggression	6.28 (1.99)	5.99 (1.44)	6.67 (2.52)	6.35 (2.30)	6.03 (1.49)	6.69 (2.87)	6.18 (1.56)	5.96 (1.39)	6.64 (1.77)
Reactive overt aggression	6.59 (2.76)	6.17 (2.39)	7.19 (3.12)	7.16 (3.31)	6.76 (3.08)	7.57 (3.49)	5.95 (1.75)	5.66 (1.38)	6.55 (2.22)
Reactive relational aggression	6.85 (2.01)	6.67 (1.62)	7.12 (2.44)	7.13 (2.39)	6.92 (1.87)	7.35 (2.81)	6.53 (1.39)	6.44 (1.33)	6.70 (1.51)

Note: Total refers to both male and female participants within each sample. Values are taken from the 20-item four-factor measurement model. Values are presented as M (SD).

## References

[B1-behavsci-16-01283] Vagos P., Rodrigues P. F. S., Pandeirada J. N. S., Marsee M. A. (2025). Aggressive behavior in adolescents and emerging adults: The psychometrics of the Portuguese brief peer conflict scale (Brief-PCS). Behavioral Sciences.

