# Supplementary material for: Aggressive Behavior in Adolescents and Emerging Adults: The Psychometrics of the Portuguese Brief Peer Conflict Scale (Brief-PCS)
Source: Behav Sci (Basel). 2025 Oct 10;15(10):1378. doi: 10.3390/bs15101378 (PMC12561357; doi:10.3390/bs15101378)
Supplement: Supplementary file 1 [file behavsci-15-01378-s001.zip › behavsci-3687302-supplementary.pdf]

Supplementary Material

Figure S1: Item loading values and spearman correlation values between measures taken from Model 2: 20-item four-factor forms of aggression model.

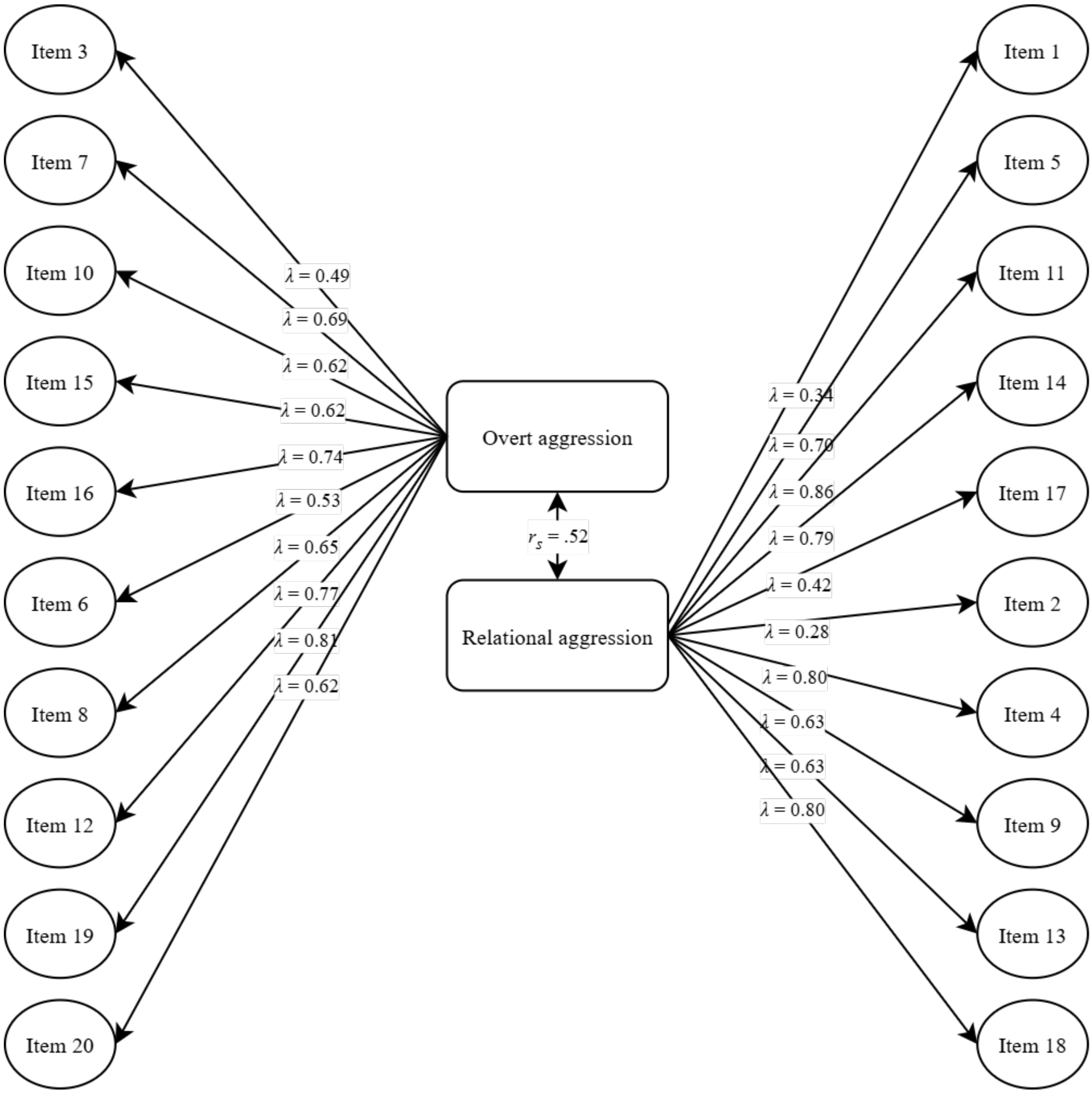

Note: All loading and correlation values were significant at $p < .001$.
